# Safety analysis of Epzicom® (lamivudine/abacavir sulfate) in post-marketing surveillance in Japan†

**DOI:** 10.1002/pds.3588

**Published:** 2014-03-03

**Authors:** Tomoko Kurita, Tomomi Kitaichi, Takako Nagao, Toshiyuki Miura, Yoshifumi Kitazono

**Affiliations:** 1 ViiV Healthcare K.K.Tokyo, 151-8566, Japan; 2 Department of Clinical Medicine, Institute of Tropical Medicine, Nagasaki UniversityNagasaki City, Nagasaki Prefecture, Japan

**Keywords:** abacavir, lamivudine, human immunodeficiency virus, hypersensitivity, myocardial infarction, Japan, pharmacoepidemiology

## Abstract

**Purpose:**

To obtain safety and effectiveness data on a combined anti-HIV drug, Epzicom (abacavir 600 mg/lamivudine 300 mg), a post-marketing surveillance on Epzicom that was required by the Japanese regulatory authority was conducted between January 2005 and December 2010.

**Methods:**

A joint survey (HIV-related drug [HRD] survey) has been conducted involving manufacturers of drugs for treatment of HIV infection in Japan. Safety and effectiveness data from total 624 cases (1107.3 person-years) registered to the HRD surveys and received Epzicom were obtained. Adverse drug reactions (ADRs) were defined as adverse events (AE) of which association with Epzicom could not be ‘ruled out’.

**Results:**

It was found that the incidence of ADR was 32.4% (202/624 cases) on the case basis. In addition, the frequently reported ADR included hyperlipidaemia (59 cases), hypertriglyceridaemia (21 cases), blood bilirubin increased (19 cases), gamma-glutamyltransferase increase (14 cases), blood triglyceride increase (14 cases) and rash (14 cases). Serious AEs were seen in 19 patients (30 events), including one death (no evident association with Epzicom). There were four cases (0.6%) of survey-defined ‘hypersensitivity’, and the incidence was 0.9% (4/445) among abacavir naïve patients; none of which was reported as serious. No case of myocardial infarction was reported. One pregnant case who delivered a normal baby by caesarean section was reported to have experienced aggravation of anaemia and nausea.

**Conclusions:**

The post-marketing surveillance indicated that the incidence of both ischaemic heart disease and hypersensitivity associated with Epzicom was considerably low, suggesting that this drug can be safely used in the Japanese population.

## INTRODUCTION

In recent years, prognosis of human immunodeficiency virus type 1 (HIV-1) infection has been markedly improved by combinational antiretroviral therapy (cART). It is no exaggeration to say that adherence to cART is the most important critical factor to determine success/failure of this therapy. The cART regimen recommended for initial treatment includes two nucleoside reverse transcriptase inhibitors (NRTIs) as the backbone, in combination with a single non-NRTI (NNRTI) or a protease inhibitor or an integrase inhibitor.^1^–^4^

Epizicom is a fixed-dose combination tablet developed by GlaxoSmithKline, containing 300 mg of lamivudine and 600 mg of abacavir, for the purpose of improving adherence to drug intake and enabling once-daily oral administration of the backbone (one tablet/dose). The combination of abacavir +lamivudine has been used in many overseas clinical studies to show its effectiveness against HIV infection,^5^–^10^ and it is listed as a favourable NRTI backbone combination in international and local HIV treatment guidelines as of 2012.^2^–^4^ Because this combined drug was approved in the USA in August 2004 and in Europe in December 2004, it has been approved in more than 40 countries by March 2011. In Japan, it was marketed in January 2005. The application for approval of Epzicom in Japan was subjected to preferential review as anti-HIV drug, and its review by the regulatory authority was made quickly on the basis of overseas data. Its components abacavir (Ziagen®) and lamivudine (Epivir®) have been marketed in Japan since 1999 and 1997, respectively. Thus, there had already been much clinical experience as to the safety and efficacy of long-term use of the components of Epzicom in Japan by the time of submission. However, no domestic clinical data on the combined form were available before approval of the product. Thus, the approval for this product in Japan was made with a condition that the marketing authorization holder collects information on clinical use of this product within the framework of post-marketing surveillance. In response to this special requirement, post-marketing data on clinical use of Epzicom were collected from 624 cases managed at 27 domestic facilities between January 2005 and December 2010. We here report safety and effectiveness data on this product obtained from the post-marketing surveillance. The safety data presented here is based on those officially reported to the Japanese regulatory authority (Pharmaceuticals and Medical Devices Agency [PMDA]) on 22 March 2011.

## METHODS

### Subjects

In Japan, anti-HIV drugs are designated orphan drugs (medicines used for rare diseases). Because the number of HIV + people is small in Japan and anti-HIV drugs are commonly used with other drugs for treatment of HIV infection (anti-HIV drugs, drugs for treatment of opportunistic infection and so on), a joint survey (HIV-related drug [HRD] surveys) has been conducted, involving manufacturers and distributors of drugs for treatment of HIV infection. The survey has been conducted at registered sites, designed to collect safety and effectiveness information on antiretrovirals marketed in Japan. It was designed to enrol all of the patients receiving antiretrovirals in principle; however, the decisions of the enrollment were made by contractor physicians; a common survey form has been filled out by the contractor physicians and recollected in each year. The management of the survey has been delegated to Nihon Ultmarc Inc. (currently CMIC-PMS Co., Ltd.). The post-marketing survey on Epzicom covered all Epzicom-treated patients registered to the HRD survey between January 2005 and March 2009 (final follow-up in December 2010). Note that the safety data presented here are based on those officially reported to the PMDA on 22 March 2011 after the completion of mandatory post-marketing surveillance for this drug. In addition, a survey on safety of Epzicom use during pregnancy was conducted if such cases were experienced.

### Observed items for safety analysis

Information was collected as to the reason (disease name) for use of the product; gender; age; race; history of anti-HIV drug treatment; complications upon registration (including renal dysfunction, hepatic dysfunction, and haemophilia); concomitant drugs; CDC disease stage^11^ situations of product use (dosing method, dose level, dosing period); situation of concomitant drug use; presence/absence of adverse events (AEs) developing after the start of Epzicom treatment; and name of AEs and their date of onset, course, intervention and seriousness (events causing death, disabilities, hospitalization, semi-serious outcome, congenital anomalies, etc., listed in Item 253 of the Pharmaceutical Affairs Law Enforcement Rules were deemed as serious). The causal relationship of each AE to the product was rated on a five-category scale ‘definitely associated’, ‘associated’, ‘not ruled out’, ‘unknown’ or ‘ruled out’. The AEs except ‘ruled out’ were defined as ‘adverse drug reactions (ADR)’ in the present analysis. Terminology of AEs in this report strictly follows MedDRA version 13.1. In addition, the following were investigated as topics of special focus: (1) hypersensitivity reaction (criteria for hypersensitivity given in Table 1); (2) association with pre-existing hepatic dysfunction; and (3) Epzicom use in pregnant women.

**Table 1:** Criteria for hypersensitivity†

Category A	Hypersensitivity/anaphylactic symptoms/allergic reactions/drug allergy
Category B	Cases meeting the following two or more items:
	• Rash
	• Fever
	• Gastrointestinal symptoms (nausea, vomiting, diarrhoea and abdominal pain)
	• Constitutional symptoms (coma, fatigue, malaise, myalgia and abnormal chest radiographs [infiltration is mainly noted and may be localized in some cases])
Exclusion criteria	• A patient in whom other causes are highly probable despite the presence of hypersensitivity-like symptoms
	• A patient without recurrence after readministration of abacavir
	• A patient with disappearance of symptoms during treatment with abacavir
	• A patient who does not meet the criteria for category B despite suspected hypersensitivity to abacavir

† Patients who meet the criteria for category A or B but not the exclusion criteria are determined to have hypersensitivity to abacavir.

### Effectiveness analysis

To investigate effectiveness of the drug, surrogate markers (plasma HIV-RNA copy number and CD4 +T-cell count) were measured before and after starting Epzicom treatment; however, this analysis was limited to the patients who had never received prior antiretroviral treatment.

### Statistical analysis

Background variables of patients were subjected to stratified analysis and so on, using chi-square test or Fisher's exact test, and *p* < 0.05 (two-tailed) was regarded statistically significant. For multivariate analysis, logistic regression analysis was performed with stepwise selection. All of the statistical analyses were conducted using SAS Ver 9.13.

## RESULTS

### Background characteristics of the surveyed patients

The survey form was recollected from all of the 624 patients treated with Epzicom among the patients registered to this survey from nationwide 27 facilities participating in the joint HRD survey. Analysis of safety was conducted on 1107.3 person-years, and 126 of 624 were lost during the observed period. Table 2 summarizes the background characteristics of the patients. Of the 624 patients surveyed, 94.4% (589 cases) were Japanese, with the percentage of men being 92.8% (579 cases). Age ranged from 10 to 81 years, with the percentage of adults (15 ≤ and ≤ 64 years) being 96.0% (599 cases) and the percentage of elderly patients (over 65 years) being 3.7% (23 cases). The reason for use of this product was HIV infection in all cases, with the mean daily dose level being one tablet in all cases. The number of anti-HIV drugs concomitantly used was one in 45.5% (284 cases) and two in 42.1% (263 cases). Thus, all cases were treated with two or more drugs, including Epzicom, implying that all patients were treated with three or more anti-HIV agents. The percentage of patients without a history of other anti-HIV drug therapy before the start of Epzicom therapy (treatment-naïve) was 34.1% (213/624).

**Table 2:** Characteristics of the subjects

		Safety analysis population	
Patient factor		Patients (*N*)	Proportion (%)
Total		624	100.0
Reason for use	HIV infection	624	100.0
Sex	Male	579	92.8
	Female	45	7.2
Age†	15–64 years	599	96.0
	65–81 years	23	3.7
Ethnic groups	Japanese	589	94.4
	Others	35	5.6
History of treatment with antiretrovirals	Absent	213	34.1
	Present	411	65.9
History of allergy	Absent	322	51.6
	Present	210	33.7
	Unknown	92	14.7
Complications	Absent	158	25.3
	Present	466	74.7
Renal impairment	Absent	586	93.9
	Present	38	6.1
Hepatic disorder	Absent	461	73.9
	Present	163	26.1
Haemophilia	Absent	584	93.6
	Present	40	6.4
Concomitant use of non anti-HIV drugs	Absent	0	0.0
	Present	624	100.0
Number of concomitant anti-HIV drugs‡	None	0	0.0
	1 drug	284	45.5
	2 drugs	263	42.1
	3 drugs	62	9.9
	≥4 drugs	15	2.4
CDC classification	A	240	38.5
	B	34	5.4
	C	121	19.4
	Unknown	228	36.5
Total duration of treatment (days)	2–180	623	99.8
	181–365	529	84.8
	366–730	444	71.2
	731–1517	234	37.5

† Two pts were <15 years old.

‡ Total numbers throughout the observed periods.

### Data on adverse drug reactions

In this survey, ADRs were defined as AEs whose causal relationship to the product could not be ‘ruled out’. As shown in Table 3, 202 of the 624 patients showed a total of 325 ADRs, with the incidence being 32.4% (202/624). In addition, ADRs whose causal relationship to the product was rated as ‘definitely associated’ or ‘associated’ were seen in 58 patients (9.0%, 101 events). In analysis of the incidence of ADR by MedDRA system organ class, the incidence was the highest with ‘metabolism and nutrition disorders’ (13.9%, 87/624 patients), followed by ‘investigations (laboratory abnormality)’ (10.3%, 64/624), ‘gastrointestinal disorders’ (4.3%, 27/624), ‘skin and subcutaneous tissue disorders’ (4.0%, 25/624), ‘hepatobiliary disorders’ (3.7%, 23/624), ‘psychiatric disorders’ (1.3%, 8/624) and ‘nervous system disorders’ (1.3%, 8/624). Regarding the names of ADR, the expressions used by the reporting physicians were converted into MedDRA preferred terms, thereby separately processing the ADRs of different expressions used by reporting physicians even if they looked similar (e.g. abnormal hepatic function vs liver disorder, and rash vs drug eruption). When analysed in this way, the number of reported cases was the largest with hyperlipidaemia (59 cases), followed by hypertriglyceridaemia (21 cases), blood bilirubin increased (19 cases), gamma-glutamyltransferase increase (14 cases), blood triglyceride increase (14 cases), rash (14 cases), hyperuricaemia (eight cases), hyperbilirubinaemia (eight cases), blood uric acid increase (eight cases), hepatic dysfunction (seven cases), liver disorder (seven cases), nausea (seven cases), drug eruption (five cases), hypertension (five cases), diabetes mellitus (five cases), diarrhoea (five cases) and so on. Serious AEs were reported on 19 patients (30 events), including two cases each of pancreatitis acute, fever, liver disorder and drug eruption, and one case each of other serious AEs. Of these serious AEs, two events seen in two patients were reported as ‘associated’ with Epzicom (hepatic dysfunction and immune reconstitution inflammatory syndrome). There was one fatal case (pancytopenia), and the causal relationship to Epzicom was reported as ‘not ruled out’; this patient had syphilis, esophageal candidiasis and HIV encephalopathy as underlying diseases, and presented with bone marrow suppression under Valgancyclovir and AZT/3TC prior to starting Epzicom.

**Table 3:** Adverse drug reactions observed during the treatment with Epzicom† (325 events in 202 subjects)

Adverse drug reaction	Cases (%)
**Blood and lymphatic system disorders**	**4** (**0**.**64**)
Iron deficiency anaemia	1
Normochromic normocytic anaemia	1
Pancytopenia	1
Haemorrhagic diathesis	1
**Cardiac disorders**	**3 (0.48)**
Atrioventricular block complete	1
Atrioventricular block first degree	1
Cardiac failure	1
**Endocrine disorders**	**1 (0.16)**
Hyperthyroidism	1
**Gastrointestinal disorders**	**27 (4.33)**
Abdominal discomfort	2
Abdominal pain	1
Abdominal pain upper	2
Ascites	1
Constipation	1
Diarrhoea	5
Gastritis	2
Gingivitis	1
Nausea	7
Pancreatitis acute	2
Reflux oesophagitis	2
Vomiting	1
Abdominal symptom	1
**General disorders and administration site conditions**	**7** (**1**.**12)**
Asthenia	1
Malaise	3
Pyrexia	3
**Hepatobiliary disorders**	**23 (3.69)**
Cholelithiasis	1
Hepatic function abnormal	7
Hepatitis fulminant	1
Hyperbilirubinaemia	8
Jaundice	1
Liver disorder	7
**Immune system disorders**	**1 (0.16)**
Immune reconstitution symdrome	1
**Infections and infestations**	**6 (0.96)**
Hepatitis C	2
Herpes zoster	3
Influenza	1
Atypical mycobacterial infection	1
**Injury, poisoning, and procedural complications**	**2 (0.32)**
Spinal compression fracture	1
Lumbar vertebral fracture	1
**Investigations**	**65** (**10**.**42**)
Alanine aminotransferase increased	2
Aspartate aminotransferase increased	2
Blood bilirubin increased	19
Blood cholesterol increased	3
Blood creatine phosphokinase increased	1
Blood creatinine increased	1
Blood glucose increased	1
Blood lactate dehydrogenase increased	1
Blood triglycerides increased	14
Blood uric acid increased	8
Gamma-glutamyltransferase increased	14
Glucose urine present	1
Blood urine present	1
Haemoglobin decreased	1
Liver function test abnormal	3
Low density lipoprotein increased	1
Platelet count decreased	4
Lymphocyte count increased	1
Neutrophil count decreased	1
White blood cell count decreased	1
Blood alkaline phosphatase increased	4
**Metabolism and nutrition disorders**	**87 (13.94)**
Diabetes mellitus	5
Glucose tolerance impaired	1
Gout	1
Hypercalcaemia	1
Hypercholesterolaemia	3
Hypertriglycaeridaemia	21
Hyperuricaemia	8
Metabolic disorder	1
Hyperphosphatasaemia	1
Decreased appetite	1
Hyperlipidaemia	59
**Musculoskeletal and connective tissue disorders**	**3 (0.48)**
Myalgia	1
Osteonecrosis	1
Osteoporosis	1
**Neoplasms benign, malignant, and unspecified (including cysts and polyps)**	**2 (0.32)**
Kaposi's sarcoma	1
Castleman's disease	1
Metastatic gastric cancer	1
**Nervous system disorders**	**8 (1.28)**
Cerebral infarction	1
Disturbance in attention	1
Dizziness	2
Dysgeusia	1
Headache	2
Tremor	1
**Psychiatric disorders**	**8 (1.28)**
Depressed mood	1
Depression	3
Initial insomnia	1
Insomnia	3
Abnormal behaviour	1
**Renal and urinary disorders**	**5 (0.8)**
Calculus urinary	1
Nephrolithiasis	1
Renal impairment	3
**Respiratory, thoracic, and mediastinal disorders**	**2 (0.32)**
Cough	1
Dyspnoea	1
**Skin and subcutaneous tissue disorders**	**25 (4.01)**
Dermatitis	1
Drug eruption	5
Erythema nodosum	1
Pruritus	2
Rash	14
Rash generalized	1
Seborrhoeic dermatitis	1
Facial wasting	1
**Vascular disorders**	**5 (0.80)**
Hypertension	5

† The terms are based on MedDRA version 13.1.

Two cases of children received Epzicom (aged less than 15 years); however, no ADRs were reported.

### Ischaemic heart diseases

Although the association of abacavir with myocardial infarction (MI) was previously reported,^12^ no ischaemic heart disease (IHD) associated with abacavir sulfate (ABC) administration (including MI and angina pectoris) was reported (one MI and one angina pectoris case were reported as ABC non-related AE). In the post-marketing surveillance of Ziagen (abacavir sulfate 300 mg) conducted from September 1999 to September 2009 (enrollment of the last patients was March 2008), none of the 643 patients (1345.7 person-years) developed IHD including MI (unpublished data, submitted elsewhere). In the present surveys, no case of IHDs including MI was reported either. Note that 180 of the 624 had been involved in Ziagen post-marketing surveillance as well; therefore, when combining data from two surveys, no case of IHD was reported in total 1087 patients (2452.99 person-years).

### Adverse drug reactions by background characteristics of the subjects

The influence of the background characteristics of the patients on ADR was analysed. The analysis revealed statistically significant differences in the incidence of ADR depending on history of anti-HIV drug therapy, history of allergy, other complications and the number of anti-HIV drugs concomitantly used (Table 4), and all of which were significant with multivariate analysis (Table 5). Although effect of pre-existing hepatic dysfunction was one of the topics of special focus because ABC is eliminated primarily by hepatic metabolism, no association was observed between pre-existing hepatic dysfunction and the incidence of ADR (Table 4).

**Table 4:** The frequency of adverse drug reactions according to the characteristics of the subjects

Factors		No. of patients	With ADRs	No. of ADR events	Incidence of ADR (%)	*χ*‡ or Fisher's exact test (based on cases)
Overall		624	202	325	32.4	–
Sex	Male	579	191	311	33.0	NS
	Female	45	11	14	24.4
Age	Child	2	0	0	0	NS
	Adult†	599	198	320	33.1
	Elderly‡	23	4	5	17.4
Ethinic groups	Japanese	589	191	304	32.4	NS
	Others	35	11	21	31.4
History of treatment with antiretrovirals	Absent	213	83	132	39.0	*p* = 0.015*
	Present	411	119	193	29.0
History of allergy	Absent	322	80	129	24.8	*p* <0.0001**
	Present	210	92	148	43.8
	Unknown	92	30	48	32.6	
Complications	Absent	158	39	53	24.7	*p* = 0.018*
	Present	466	163	272	35.0
Renal impairment	Absent	586	186	299	31.7	NS
	Present	38	16	26	42.1
Hepatic disorder	Absent	461	142	213	30.8	NS
	Present	163	60	112	36.8
Haemophilia	Absent	584	189	300	32.4	NS
	Present	40	13	25	32.5
Number of concomitant anti-HIV drugs§	1 drug	284	78	126	27.5	*p* = 0.019*
	2 drugs	263	89	131	33.8
	3 drugs	62	27	46	43.6
	4 drugs ≤	15	8	22	53.3
CDC classification	A	240	84	141	35.0	NS
	B	34	11	17	32.4
	C	121	47	73	38.8
	Unknown	228	60	94	26.3	

NS, no statistical significance.

† ≥15 to ≤64 years.

‡ Elderly patients.

§ Total numbers throughout the observed periods.

* *p* < 0.05.

** *p* < 0.01.

**Table 5:** The background characteristics that are associated with the frequency of ADR^†^

Factors	Odds ratio	95% confidence interval
History of treatment with antiretrovirals	0.52	0.36–0.76
History of allergy	2.01	1.40–2.88
Complications	1.80	1.15–2.81
Number of concomitant anti-HIV drugs	1.36	1.10–1.69

For history of allergy, ‘unknown’ was regarded as ‘absent’; for CDC classification, ‘unknown’ was regarded as ‘category A’

The incidence of ADR in the group without history of anti-HIV drug therapy (treatment naïve [TN]) was 39.0% (83/213), which was significantly higher than those having history of anti-HIV drug therapy (treatment experienced [TE]) (29.0%, 119/411) (*p* < 0.05). When analysed by system organ class, the incidence of ‘skin and subcutaneous tissue disorders’ was significantly higher in TN (9.39%, 20/213) than in TE (1.22%, 5/411) (*p* < 0.0001), which was mainly driven by the larger number of cases developing rash in TN (13 cases) than in TE (one case). A possible explanation for these differences is that many of the TE patients had received component of Epzicom, namely Ziagen (abacavir) and/or Epivir (lamivudine) prior to Epzicom. The incidence of ‘metabolism and nutrition disorders’ such as hyperglycaemia, hypertriglycaeridaemia was higher among TN patients (data not shown), suggesting the possibility that in TE patients, these disorders had already been induced by anti-HIV drugs and were counted as complications at the start of Epzicom. The incidence of ADR was 43.8% (92/210) in the group having history of allergy, which was significantly higher than in those without history of allergy (24.8%, 80/322) (*p* < 0.01), which seemed to have been mainly driven by ‘rash’ (13/210 vs 4/322, *p* = 0.004). The incidence of ADRs became higher as the number of anti-HIV drugs concomitantly used increased recording 27.5% (78/284), 33.8% (89/263), 43.5% (27/62) and 53.3% (8/15) when the number of concomitant anti-HIV drugs was one, two, three and four, respectively (*p* < 0.05), suggesting the influence from multiple-drug-combined therapy.

### Hypersensitivity

Onset of hypersensitivity reaction was investigated on the basis of the survey forms filled by the physicians, using the criteria in Table 1. Four cases of survey-defined hypersensitivity were reported (Table 6), with the incidence of 0.64% (4/624), all of whom were abacavir naïve patients. Therefore, when calculating the incidence limiting to the abacavir naïve patients, the incidence was 0.9% (4/445). Close association of HLA-B*5701 with abacavir-induced hypersensitivity reaction has been described,^12^–^20^ which rarely expressed in Japanese people.^21^ Nevertheless, in the present survey, all of the patients who developed hypersensitivity were Japanese. The symptoms of hypersensitivity were ‘rash’, ‘malaise + vomiting’, ‘fever + rash’ and ‘drug eruption’ (one case each). All of these symptoms were non-serious and disappeared or improved.

**Table 6:** Patients presented hypersensitivity

	Adverse reaction	Association	Severity	Outcome	Time to onset (days)	Sex	Age	Complications	Concomitant suspected products	Comment from physicians
1	Rash	Associated	Not serious	Ameliorated	61	Male	28	Factor IX deficiency, Hepatitis C, Atrial septal defect, Pulmonary hypertension	–	
2	Vomiting, Malaise	Not ruled out	Not serious	Recovered	152	Female	32	Oesophageal candidiasis, Acute lymphocytic leukaemia	–	
3	Rash, Pyrexia	Not ruled out	Not serious	Recovered	9	Male	46	Hepatitis B	LPV/rtv	Association with HIV infection, LPV/rtv and Epzicom cannot be ruled out.
4	Drug eruption	Not ruled out	Not serious	Ameliorated	13	Male	38	–	LPV/rtv	Association with HIV infection, LPV/rtv and Epzicom cannot be ruled out.

ddI, didanosine; d4T, stavudine; AZT, zidovudine; NVP, nevirapine; 3TC, lamivudine; LPV/rtv, lopinavir/ritonavir; TDF, tenofovir disoproxil fumarate; EFV, efavirenz.

### Duration of Epzicom treatment prior to onset of adverse drug reactions

Among the 202 patients having developed ADR (323 reactions in total), the duration of Epzicom treatment before onset of ADR was known in 187 patients. After the start of Epzicom, ADR developed within 180 days in 63.9% (129/202), between 181 and 365 days (1 year) in 78.2% (158/202), and on Day 366 and later in 14.4% (29/202). The frequent ADRs occurred within 180 days were hyperlipidaemia (25 cases), rash (14 cases), blood bilirubin increased (14 cases), hypertriglyceridaemia (13 cases) and so on. The most frequent ADRs developed on Day 366 and later was hyperlipidaemia (15 cases).

### Pregnant cases

Information on pregnant women was collected on one case during the survey period (with one newborn collected as well). In this case, non-serious ‘aggravation of anaemia’ (56 days after the start of Epzicom) and ‘aggravation of nausea’ (28 days after the start of Epzicom) developed during the course of pregnancy (Epzicom had been started 142 days before the delivery). The causal relationship to the product was not ‘ruled out’. A normal baby was delivered at gestational age of 36 weeks by a caesarean section. Mild anaemia and transient tachypnea were reported in the neonate on the date of birth.

### Effectiveness analysis

**Figure 1:**
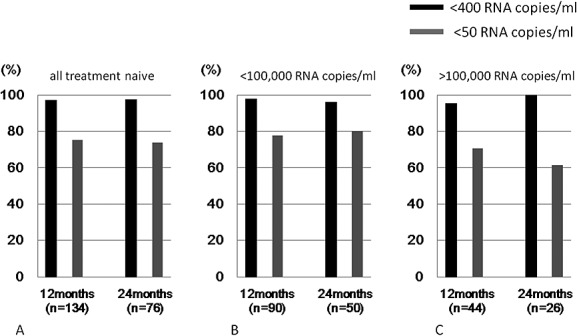
Proportion of treatment-naïve subjects who achieved <50 or <400 HIV-RNA copies/mL after 12 and 24 months of treatment with ABC/3TC containing regimen. (a) All treatment-naïve HIV + subjects, (b) treatment-naïve subjects with baseline pVL <100 000 RNA copies/mL, and (c) treatment-naïve subjects with baseline pVL >100 000 RNA copies/mL. Note that the denominators are those who were on Epzicom treatment for the given duration, therefore not including those who stopped Epzicom earlier

**Figure 2:**
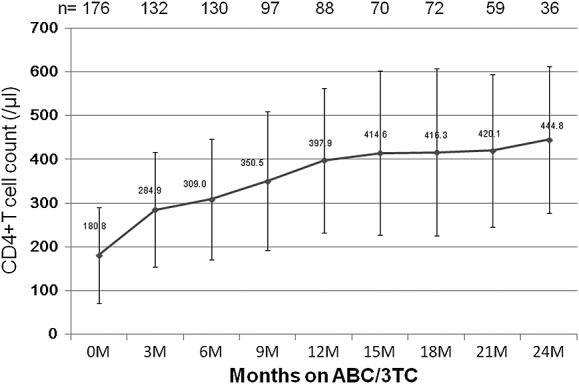
Change in mean CD4 + T-cell count after initiation of ABC/3TC in treatment-naïve HIV + subjects. Mean absolute CD4 + T-cell count is shown in the solid line, and the vertical bars indicate standard deviation. Note that the number of subjects shown is those whose CD4+ T-cell count was available at each time point

Effectiveness analysis was performed on only TN patients whose HIV surrogate markers (plasma viral load and CD4 + T-cell count) at baseline and after the start of Epzicom were available. Among the TN patients who received therapy including Epzicom for 12 consecutive months, the percentages of patients with plasma HIV-RNA <400 copies/mL and <50 RNA copies/mL were 97.5% (130/134) and 74.6% (101/134), respectively. In addition, among the TN patients treated for 24 consecutive months, the percentages were 97.5% (74/76) and 73.7% (56/76), respectively (Figure 1a). When the patients were stratified according the baseline HIV-RNA level (cutoff level: 100 000 copies/mL), plasma viral load at 12 and 24 months was <400 copies/mL in the majority cases, whereas the percentage of cases <50 RNA copies/mL was lower regardless of the baseline viral load (<100 000 RNA copies/mL, 77.8% [12 months], 80.0% [24 months]; >100 000 RNA copies/mL, 70.5% [12 months], 61.5% [24 months]) (Figure 1b and 1c). The mean CD4 + T lymphocyte count increased from 180/μL (baseline) to 444/μL after 24 months of the Epzicom treatment (Figure 2).

## DISCUSSION

On the basis of the 10-year post-marketing survey on Ziagen Tablet (abacavir sulfate 300 mg), we recently reported that the incidence of serious ADR associated with abacavir was 1.9% (12/643) and that no death occurred from the use of this drug, indicating abacavir can be used relatively safely for Japanese HIV-positive patients^22^. Lamivudine has been marketed for over 10 years in Japan and used not only for HIV infection but also for Hepatitis B virus infection,^23^–^25^ showing superior tolerability. The present post-marketing surveillance on Epzicom Tablet (a combination of abacavir and lamivudine) in Japanese patients was expected to yield more useful information, particularly for abacavir.

The incidence of ADR reported in this survey was clearly lower than those in the post-marketing survey on Ziagen tablet. The most feasible explanation for this was because substantial part of the patients had received Ziagen (abacavir) or Epivir (lamivudine) before starting Epzicom, few of them presented new ADRs upon switching to Epzicom. A couple of other factors might have contributed to this finding, including ‘trend toward early diagnosis and initiation of cART’ before patients becoming sick. Likewise, improved safety profile of other anti-HIV drugs concomitantly used might have affected the findings.

A recent meta-analysis by the US FDA denied the possibility that abacavir is associated with MI.^26^ However, this possibility is one of the major reasons why the combination abacavir/lamivudine has not been listed as a preferred NRTI backbone for TN patients in the DHHS Guidelines.^1^ The incidence of IHD is known to be lower in Japanese people than in Western people,^27^–^29^ yet the possibility of IHD is sometimes taken into account when physicians prescribe abacavir to Japanese patients. In the present survey, analysis of the data combined from post-marketing surveys on Ziagen Tablet^22^ and Epzicom Tablet revealed no case developing IHD among 1087 patients treated with abacavir (2452.99 person-years). This result seems to serve as information useful in prescription of this product in Japanese population. However, regular pharmacovigilance activity is of paramount importance to monitor any signal for the risk of MI by this drug for patient safety.

In the post-marketing survey on Ziagen, 15 cases of hypersensitivity (2.3%) were reported, whereas the incidence of hypersensitivity was only 0.9% (4/445) in this Epzicom survey, which reassures that the risk of hypersensitivity to abacavir in Japanese population is substantially low; nevertheless, in view of the report on cases of suspected hypersensitivity among patients not expressing HLA-B*5701,^13^ it is necessary to monitor patients carefully when Epzicom is used.

Regarding effectiveness, because ACTG5202 Study demonstrated that abacavir/lamivudine (ABC/3TC) is inferior to tenofovir/emtricitabine (TDF/FTC) in patients with baseline virus load over 100 000 RNA copies/mL,^6^ it has been one of the reason why the DHHS guideline does not list ABC/3TC as preferred NRTI backbone.^1^ However, other studies reported no difference in effectiveness between the two arms.^5^,^30^,^31^ In the present survey, TN patients with baseline plasma virus load over 100 000 copies/mL showed relatively good virological responses; however, we could not make any definite conclusion because it was non-controlled observational study designed for safety surveillance. Although data generation on effectiveness of ABC/3TC when used with modern powerful key drugs such as boosted darunavir or raltegravir has been evolving,^32^,^33^ evidence from controlled clinical trials should be generated for the fair evaluation for the effectiveness of Epzicom.

In conclusion, the present survey convinced abacavir and lamivudine can be used relatively safely in Japanese HIV-positive population. However, regular pharmacovigilance should be continued for patient safety.

## CONFLICT OF INTEREST

Tomoko Kurita, Tomomi Kitaichi, Takako Nagao, Toshiyuki Miura and Yoshifumi Kitazono are employees of ViiV Healthcare K. K.

## KEY POINTS

- Abacavir/lamivudine fixed-dose combination tablet can be well tolerated by Japanese HIV + people.
- Hypersensitivity reaction to abacavir was observed in 0.9% of treatment-naive HIV + patients.
- No case of myocardial infarction associated with abacavir administration was observed among 624 cases (1107.3 person-years).

## ETHICS STATEMENT

This was a surveillance mandated by the Japanese regulatory authority to collect information from all of the patients receiving the drug. IRB approval were obtained where appropriate according to institutional rule.
